# Study on the Influence of Proprioceptive Control versus Visual Control on Reaction Speed, Hand Coordination, and Lower Limb Balance in Young Students 14–15 Years Old

**DOI:** 10.3390/ijerph181910356

**Published:** 2021-10-01

**Authors:** Dan Alexandru Szabo, Nicolae Neagu, Silvia Teodorescu, Ciprian Marius Panait, Ioan Sabin Sopa

**Affiliations:** 1 Department ME1—Faculty of Medicine in English, George Emil Palade University of Medicine, Pharmacy, Science, and Technology of Targu Mures, 540139 Targu Mures, Romania; 2 Department of Human Movement Sciences, George Emil Palade University of Medicine, Pharmacy, Science, and Technology of Targu Mures, 540139 Targu Mures, Romania; nicolae.neagu@umfst.ro; 3 Department of Doctoral Studies, National University of Physical Education and Sports, 060057 Bucharest, Romania; silvia.teodorescu@unefs.ro; 4 Department of Physical Education and Sports, National University of Physical Education and Sports, 060057 Bucharest, Romania; cipi.rapid@yahoo.com; 5 Department of Environmental Sciences, Physics, Physical Education and Sports, “Lucian Blaga” University Sibiu, 550012 Sibiu, Romania; sabin.sopa@ulbsibiu.ro

**Keywords:** proprioceptive control, visual control, reaction time, hand coordination

## Abstract

Currently, sports activities require a high reaction speed, coordination, and balance, highlighting the relationship between proprioceptive control, visual control, and hand–eye coordination in youth. The present research assessed the proprioceptive control, reaction speed, and lower limb balance of youth from five different schools to identify the level of physical preparation of children in this direction. This prospective study was conducted between 1 January 2020 and 29 February 2020. A total of 107 healthy children (33 females and 74 males) with appropriate medical conditions, aged between 14 and 15 years, from five Romanian schools were included in the experiment. All children were assessed for visual control and reaction speed with the ruler drop test, and for lower limb balance, the standing stork test was used. Statistical analysis included descriptive statistics, data series distribution, and comparison of means and medians using specific statistical programs. Comparison of medians highlighted significant statistical differences in the standing stork test with eyes closed and the dominant leg compared with the nondominant leg (*p* = 0.0057). Males were compared to females at the nondominant leg (*p* = 0.0179); closed eyes were compared with opened eyes for the nondominant leg (*p* = 0.0175 and 0.0006) for the ruler drop test comparing the dominant hand with the nondominant hand (*p* = 0.0212). Children who engage in sports activities better integrated sensory information in motor action execution based on reaction speed and coordination with the nondominant hand.

## 1. Introduction

In general, practicing sports activities and physical exercise helps develop motor abilities such as balance, reaction time, proprioceptive, and visual control. The ability to retain body stability plays a crucial role in infants’ growth, and it is a fundamental prerequisite for competent and nuanced motor skills [1,2].

Studies that independently test static and dynamic balance capabilities are widely available, but few studies directly tested the relationship between static and dynamic balance capabilities and functional balance, particularly for single-leg balance [3,4,5].

Proprioceptive control is essential for improving sports performance, medical disorders, everyday life activities, or further sports performance. Some specialists associated proprioceptive control with somatosensory or proprioceptive deficiencies with effects on movement regulation that can develop from early ages [6,7,8], being linked with a low level of movement and sports practice. These capacities were associated with the sense of relative location and movement of the limbs and body [9], needed in any human activity. Other proprioceptive control opinions highlighted the idea that mechanoreceptors in the joints, muscles, tendons, and skin [10] provide proprioceptive information.

Balance and proprioceptive control are often used in neuromuscular rehabilitation programs that incorporate specific balance exercises borrowed from the area of athletic training. Developing balance and proprioceptive control from early ages in school activities can prevent the apparition of deficiencies and optimize performance, prevent injury, or provide rehabilitation [11,12,13,14].

Scientific papers have shown the effectiveness of balance and proprioceptive exercises in school and high school sports activities to reduce sport-related injury risk and enhance functional performance after sports injury [15]. It is suggested that [16] transformations in proprioception and neuromuscular management are the main reasons for these effects. However, evidence-based practice in school physical education programs is hampered by the large variety of exercises used for neuromuscular training programs. Other authors [17,18] characterized neuromuscular training in early childhood as multi-intervention programs with a combination of balance, strength, plyometric, agility, and physical education and sport-specific exercises, while most authors described balance and stabilization exercises.

Evaluating stability and coordination in physical education activities can provide essential information about proprioceptive and visual control and other necessary skills such as hand–eye coordination and reaction time.

Physical exercise represents the primary method in developing coordination and balance; therefore, to generate the required locomotor modifications for healthy and efficient sports activity, sensorimotor integration of visual and proprioceptive inputs is crucial [19].

Superior balancing capacity is essential in many sports to reach the maximum reasonable degree and prevent injury to the lower limbs [20,21,22]. The central nervous system incorporates optical, vestibular, and proprioceptive knowledge to monitor the balance to generate motor commands that organize muscle activity patterns [23,24,25]. Proprioception is characterized as the ability to incorporate sensory signals from different mechanoreceptors to determine the location of the body and movements in space [26,27], and it plays a crucial role in balance control [24,25,28,29,30,31]. Proprioceptive control from any part of the body influences the equilibrium skill of the entire body positively. The theory of sensory adaptation, which concludes that the central nervous system might change the dependence on more reliable data sources to improve balance control [25,26,28], implies that, for instance, wherever perception is used to monitor movement in exterior surroundings, the central nervous system can depend more around proprioceptive knowledge for balance control from specific components of the corpse. This is in favor of, for example, the ankle’s proprioception, which can be one of the more essential components to balance control in athletics, since the ankle–foot structure is the single component of the body that touches the floor during most athletic activities [29].

Postural stability of school children represents a long-discussed problem that is considered a starting point for body dysfunctions such as lordosis, scoliosis, and kyphosis deficiencies. In works that consider postural stability from the perspective of plastic surgery, various tests, conditions, and variables have been used to determine and describe the structure of postural stability. Although several authors have recommended various strategies to categorize many of the tests [32], no single classification system has been universally adopted. From a general perspective, physical exercise and sports practice are examined as two of the compelling methods of combating physical deficiencies, visual acuity, balance, hand coordination, and reaction time, contributing to the well-balanced growth of children.

There has been extensive knowledge and much evidence-based research regarding the importance of sports and motor abilities, physical health, and wellbeing [33]. In addition, more and more shreds of evidence show the benefits of sports and motor activities in terms of the level of physical development of children [34]. Therefore, the purpose of motor activities and sports is to combine these positive effects and even produce synergistic effects. In this case, experts from different fields have emphasized the benefits of sports, which often regard performing activities and the importance of their practice and wellbeing [35]. The term sports can be used interchangeably with sports activities and is based on the definition of inclusiveness. Europe (1992) describes sports as “all forms of physical activity aimed at expressing or improving physical and health through casual or organized participation, forming social relationships or achieving results in competitions at all levels” [36,37].

In addition to the health promotion effects of motor activities, sports and physical activity are related to social welfare, including young people’s internal and interpersonal development, because they provide unique opportunities for all practitioners, in our case, children. They connect individuals, other people, and themselves in a healthy lifestyle [38], thereby achieving a series of positive effects at the same time [37].

This study planned to evaluate the visual control parameters, proprioceptive control, reaction time, balance, and hand coordination, using two tests to discover children’s potential to practice sports activities compared with those who do not practice extra school sports activities. The research’s general hypothesis started from the idea that balance, hand–eye control, and proprioceptive control are essential motor abilities developed in the youth and are other essential skills in sports and everyday life. Additionally, other secondary objectives of the research were to assess the level of balance, hand–eye coordination, and proprioceptive control of children that practice performance sports compared with those that practice only mandatory physical education classes, comparing the level of development of those skills at the dominant hand vs. the nondominant hand/dominant leg vs. the nondominant leg and also comparing the results of male vs. female subjects.

Knowledge of the level of manifestation of balance and hand–eye coordination, as factors that ensure the stability of positions (postures) and the orientation of movements in space on the one hand and the person’s ability to synchronize the integration of visual stimuli with hand movements on the other can lead to the efficiency of domestic, professional, and sports activities. In developing the research, we started from the premise that coordinated movement is a type of movement that interconnects visual, vestibular, and kinesthetic analyzers and is dependent on the person’s ability to detect, perceive, and use appropriate sensory information.

The results obtained can help teachers, coaches, and athletes develop training protocols to enrich the technical repertoire in different sports branches and identify possible deficiencies that can be remedied through special exercises.

## 2. Materials and Methods

### 2.1. Study Design and Subjects

The research included a sample of *n* = 107 children (33 females and 74 males) selected from five gymnasium schools from Târgu Mureṣ, Romania, and was conducted between 1 January 2020, until 29 February 2020. Out of 107 children, we would like to mention that 47 practice sports (12 females and 35 males), and 60 (21 females and 39 males) only attend physical education classes.

The research included children between the ages of 14 and 15 years, children with a good medical condition and proper health who had no injuries for the last six months, had not received any medical treatment, and had no interruption of sports activities (injuries or any pause from physical education classes or sports performance activities). The research included children who practiced physical education in the last six months at their schools and children who practiced performance sports at a professional club with a minimum of three training sessions of one hour and a half per week. Any student younger than 14 years old and older than 15 years old with medical problems, such as an orthopedic disorder that would hinder their ability to conduct the test, was omitted from the analysis.

We want to mention that in the present research, 135 subjects were initially included, which was the number of students aged between 14 and 15 years from the mentioned educational units, of which 15 were missing at the time of testing. We did not receive informed consent signed by the parents from 13 subjects.

From the point of view of the sample size, by entering the data into the Minitab (Minitab, LLC, State College, Pennsylvania, USA) software, from the total 135 subjects without any health problems, we needed 67, or more measurements/surveys were needed to obtain a confidence level of 95% such that the real value was within ±5% of the measured/surveyed value. In contrast, we introduced –1 because we have a less than (<) alternative hypothesis. The sample size was set at 107 and the estimated value of the standard deviation at 2.7, obtaining a statistical power value of 0.854404 (85%). We determined the statistical power (SP) to detect the proper effect, and the chosen level required was at least 0.8 (80%).

The research protocol and the purpose of the experiment were explained to all participants in advance, and because it was a study that involved minors, at the beginning of the experiment, written informed consent was obtained from parents. The protocol was approved by the Review Board of Physical Education and Sports Department, University “Lucian Blaga” Sibiu (Resolution No. 24/24/09/2019), and all the procedures were carried out in compliance with the Helsinki Declaration’s requirements.

### 2.2. Procedure

The research protocol included two psychomotor evaluations: the standing stork test [39] and the ruler drop test [40]. The research goal was to ascertain the level of proprioceptive control, visual control, reaction speed, hand coordination, and children’s balance from the experiment sample (both categories, children who practice performance sports and children who practice only physical education). Both tests were demonstrated to all participants, and the youngsters were permitted to practice several times to avoid all possible errors.

The tests we used, the standing stork test and the Ruler drop test, evaluate balance and coordination and present the following psychometric properties: reliability, which refers to the consistency and the stability in the measurement. Reliability depends on the rigor of the execution of the test and the level of motivation of the individual to perform the test, the effectiveness of the test to measure the degree of content it claims to measure, and the degree of suppositions, assumptions, and judgments based on these factors. The test grades are proper and constructive, and we wanted to highlight and evaluate the children’s ability level. We used a test battery to determine leg dominance [41], and hand preference was assessed with a handedness questionnaire [42].

The stork test assesses children’s balance and proprioceptive control from both groups (children who practice performance sports and children who only practice the sports at physical education lessons). The standing stork test was selected as the primary research method for our investigation because we considered this test a relevant balance test and needed previous research to compare with. This test is standardized and does not need the use of any unique equipment, in contrast to flamingo, a EUROFIT battery sample that necessitates the use of a specific instrument. We adopted a typical procedure in our study [43,44]. This procedure required the subject under investigation to stand effortlessly on both feet with hands around the hip, lift the right leg, and place the sole of the right foot against the left kneecap. The subject was then asked to raise the heel and stand on the toes. The stopwatch started when the heel was lifted off the floor and was halted if the hand(s) slipped off the hips, the supporting foot swiveled or moved in any direction, the nonsustaining foot broke contact with the leg, or the supporting foot heel reached the floor. The subject had to attempt to maintain this position for as long as possible. The test was practiced with eyes open and eyes closed without shoes for both the strong and weak foot. Each subject performed three attempts for each of the tests (three with eyes opened and three with eyes closed), with a pause of 3 min between attempts. The best result was recorded for the statistical analysis of this study. The data measurement was performed by specialists from the physical education domain, who performed the necessary preparation for assessing the tests. For interpreting the results, the scale used by Johnson and Nelson (1979) for adolescents was used. The scale presented for males the following interpretation of results: >50 s—excellent result, 41–50 s—above-average result, 31–40 s—an average result, 20–30 s—below-average result <20 s—poor result; and for females the following interpretation: >30 s—excellent result, 23–30 s—above-average result, 16–22 s—average result, 10–15 s—below-average result <10 s—poor result.

In the second test, the ruler drop test, used for testing visual control, hand–eye coordination, and reaction speed, the standard method was used [45] and required the investigated subject to lean on a chair with hands placed in the midprone location and the elbow tensed at 90 degrees. The forearm was supported on a table with an open hand (three attempts with the dominant hand and three attempts with the nondominant hand) at the surface edge. The investigator dangled the ruler with the ruler’s 0 cm mark on the index finger. The ruler was then dropped with a randomly assigned delay time between 2 and 5 s between the two fingers without prior announcement, and the subject was asked to catch it as quickly as possible. The order of testing of each hand was randomized. Each subject had three attempts per hand, and the best performance was recorded for further statistical analysis. The resulting scale presented by Davis (2000) for adolescents was used. The scale was used at the national level in the USA, having the following result interpretation: <7.5 cm—excellent; 7.5–15.9 cm—above average; 15.9–20.4 cm—average; 20.4–28 cm—below average; >28 cm—poor result. Additionally, this investigation offered a solution reaction time calculation of d = vt + ½ at2 (where d is distance in meters, v is initial velocity equal to 0, a is acceleration due to gravity equal to 9.81 m/s^2^, and t is time in seconds).

### 2.3. Statistical Analysis

Statistical analysis included descriptive statistics (mean, median, standard deviation) and elements of inferential statistics. The Shapiro–Wilk test was applied to determine the distribution of the analyzed data series. For the comparison of means and medians of the results in both tests of the two groups (the group of children that practice performance sports and the second group that does not practice performance sport), the ANOVA test, parametric test for unpaired data; Mann–Whitney test and Kruskal–Wallis test, nonparametric test for unpaired data; and Wilcoxon test, nonparametric test for paired data, were applied. The Pearson correlation was applied to measure the power of association between variables to evaluate independent variables’ impact on dependent variables. The significance threshold was chosen at *p* = 0.05. Statistical analysis was performed using the GraphPad Prism trial version utility and Minitab to determine the required sample size and the statistical power (SP).

### 2.4. Hypothesis of the Study

#### Statistical Hypothesis

**Hypothesis** **1** **(H1).** *Equilibrium and proprioceptive control for the dominant and nondominant segments differ significantly between the two sexes.*

H0: μ1=μ2

H1: μ1≠μ2

**Hypothesis** **2** **(H2).** *The balance and proprioceptive control for the dominant and nondominant segments differ significantly between children who practice performance sports and children who practice only in compulsory physical education lessons.*

H0: μ1=μ2

H2: μ1≠μ2

**Hypothesis** **3** **(H3).** *Reaction speed and hand–eye coordination differ significantly between children who practice performance sports and children who practice only in compulsory physical education lessons.*

H0: μ1=μ2

H3: μ1≠μ2

**Hypothesis** **4** **(H4).** *Reaction speed and hand–eye coordination differ significantly between the sexes.*

H0: μ1=μ2

H4: μ1≠μ2

## 3. Results

Table 1 shows the mean of the best performance from the ruler drop test and standing stork test (eyes closed and eyes opened) for the dominant hand/nondominant hand (in the ruler drop test) and for the dominant leg/nondominant leg (in the standing stork test), and was compared by genders. We used the Mann–Whitney test to determine the median and compare the two genders (females vs. males) for values of the dominant hand/nondominant hand (in the ruler drop test) and dominant leg/nondominant leg (in the standing stork test) (at a significant value of *p* = 0.005).

After using the Mann–Whitney test, with a significance value of *p* < 0.05, we did not discover any statistically significant differences between males and females in the ruler drop test at the dominant hand (*p* = 0.0957) and the nondominant hand (*p* = 0.3297), nor when comparing the result of females to males in the standing stork test, eyes closed with the dominant leg (*p* = 0.5848), eyes opened with the dominant leg (*p* = 0.8820), and the nondominant leg (*p* = 0.2715). The only statistically significant difference between the results of the two genders was discovered when comparing the medians of the standing stork test results with eyes closed and the nondominant leg (*p* = 0.0057).

The second step of the research compared children who practice sports activities with children who do not require practice (Table 2). The Mann–Whitney test was used for comparing the means of both groups for statistical significance (with *p* = 0.05). No statistically significant results were found comparing children who practice sports activities with those who do not practice sports in the ruler drop test with the dominant hand (*p* = 0.1459), the standing stork test with eyes closed and the dominant leg (*p* = 0.8359), the nondominant leg (*p* = 0.5937), and with opened eyes with the dominant leg (*p* = 0.4899) and the nondominant leg (*p* = 0.1368). The only statistically significant result was comparing children who practice sports activities and children who do not practice sports activities in the ruler drop test at the nondominant hand (*p* = 0.0212).

The following stage of the research compared the standing stork test results with eyes closed and with eyes opened for the dominant leg and the nondominant leg. The Wilcoxon test was used for testing the statistical significance of the results, with a value of *p* = 0.05. The results showed no statistical significance difference between the standing stork test’s results (eyes closed) and the standing stork test (eyes opened) for the dominant leg (females, *p* = 0.7613; males, *p* = 0.4588) and at the nondominant leg (females, *p* = 0.0179). The only statistically significant data were found comparing the two variants of the test (closed/opened eyes) at the nondominant leg of males (*p* = 0.0179).

The following comparison was made between the results of the standing stork test (dominant leg, eyes closed and then opened; nondominant leg, eyes closed and then opened). The results presented in Table 3 and Table 4 show no statistically significant correlations at the standing stork test, dominant leg, neither with eyes closed (*p* = 0.4502) or eyes opened (*p* = 0.6935), but statistically significant differences were found at the nondominant leg both with eyes closed (*p* = 0.0175) and eyes opened (*p* = 0.0006).

**Figure 1:**
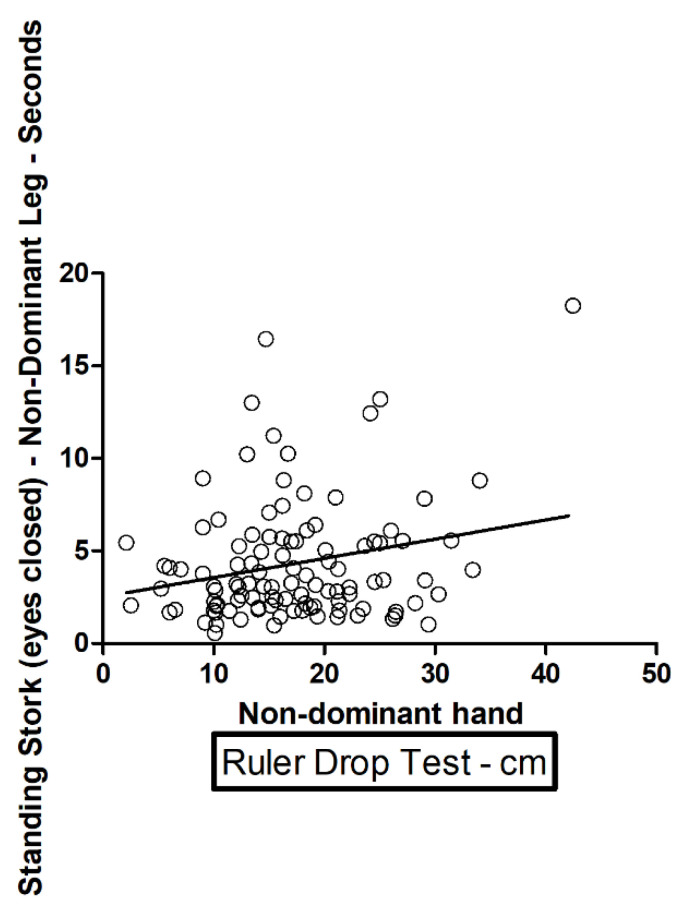
Standing stork (eyes closed) nondominant leg and nondominant hand.

**Figure 2:**
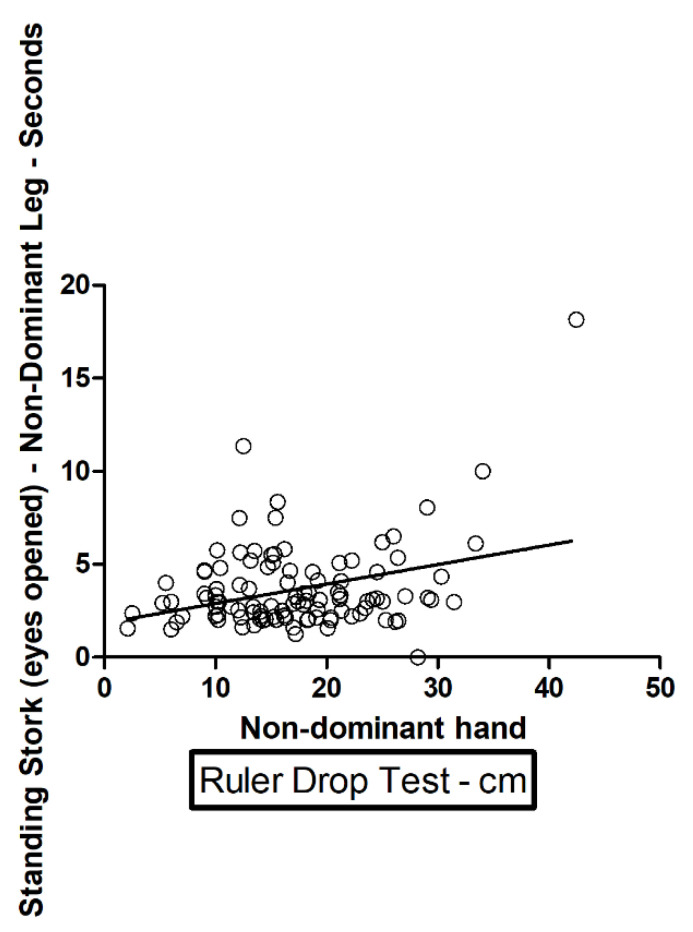
Standing stork (eyes opened) nondominant leg and nondominant hand.

A positive correlation (direct dependence) and a high value were found in the ruler drop test with the nondominant hand, correlating with a high value in the standing stork test (eyes closed, *p* = 0.0175) with the nondominant leg (Table 4 and Figure 1). The correlation is statistically significant. A positive correlation (direct dependence) with a high value in the ruler drop test with the nondominant hand, correlates with a high value in the standing stork test (eyes opened, *p* = 0.0006) with the nondominant leg (Table 4 and Figure 2). The correlation is statistically significant.

## 4. Discussion

In addition to the multitude of motor knowledge, sports activities improve proprioceptive control, hand–eye coordination, and balance skills. School environment and physical education activities proved to be efficient in developing all-around youth skills, physical conditioning, and skills such as balance, coordination, control, and precision. Our study started from the hypothesis that sports activities improve necessary skills such as balance, precision, hand–eye coordination, and proprioceptive control, which are considered necessary in youth’s general development.

The study results present a statistically significant difference (at *p* = 0.05) between children who practice performance sports and children who only practice mandatory physical education classes in the ruler drop test at the nondominant hand (*p* = 0.0212). No other significant statistical results of the ruler drop test results were found comparing males to females or the dominant hand of children who practice performance sports than those who do not practice sports activities.

Children who participate in sports performed better in the ruler drop test with their dominant hand (15.76 cm) than children who do not participate in sports (17.95 cm). However, no statistically significant difference between groups was detected at the nondominant hand as a consequence of children who participate in performance sports (15.63 cm) compared to children who do not participate in performance sports (18.41 cm). However, it is also an average result when analyzed using the interpretation scale proposed by Davis [46].

The results confirmed no statistically significant differences between gender in the stork test with eyes open and dominant and nondominant feet. Instead, statistically significant differences were found between gender at the standing stork nondominant leg with eyes closed, in favor of boys, which highlights a good proprioception level in the male gender. Our study did not record correlations between students’ age and performance in the ruler drop test and standing stork.

We found statistically significant correlations with direct dependence between a high value in the ruler drop test with the nondominant hand, which correlates with a high value in the standing stork test (eyes closed, *p* = 0.0175); nondominant leg; and a high value in the ruler drop test with the nondominant hand, which correlates with a high value in the standing stork test (eyes opened, *p* = 0.0006).

The result registered in the ruler drop test highlighted an average result of the subjects investigated (*n* = 107) at the dominant hand of 17.46 cm and the nondominant hand of 17.88 cm, measured with the scale presented by Davis [46]. The results follow the age of adolescents when motor development consolidates. Some research findings highlighted that proprioceptive development of youth between 5 and 18 develops over the years, and the level of precision improves with age. Additionally, research proves that sensory response variability decreases with age [10]. Other findings suggest that 7- to 13-year-old children flexibly use unimodal estimation information for reweighted redundant sensorimotor inputs [45]. Previous studies exploring multimodal localization indicated that 4- to 13-year-old children rely more heavily on vision when visual and proprioceptive stimuli provide the target location [45]. The same research also highlighted that proprioceptive localization changes contributed to the increased contribution of proprioception to the multisensory estimate in children aged 7–13 years, and, importantly, this result was age independent. This finding extends the results of previous studies investigating the application of multisensory integration for motor control in postural control tasks [46]. Vision was considered the dominant modality in children between 10 and 15 years old, and the output of other sensory stimuli (i.e., proprioception) increased with age until about 17–18 years [47].

Another investigation of youth balance [43] demonstrated a good association between the flamingo test and the stork test measured in 24 healthy children. The outcome showed vital significance in all correlation values, as *r*-values for the right and left leg were 0.64 and 0.56, respectively [43]. Similar to the stork test, the flamingo test also provides good results in assessing balance motor skills. Standard dynamic balance control research using static balance control of mechanical disturbances uses advanced signal processing methods such as ICA to reduce motion artifacts [48,49,50].

Relatively few static paradigms use cognitive challenges to study the neural components of balance [51,52]. In addition, except for static balance studies that use sensory challenges (such as opening and closing eyes), all other studies were conducted on healthy young people. Brain activity analysis includes disturbance-induced ERP and frequency analysis during standing balance control, which adds activation findings during balance challenges, regardless of the type of challenge (sensory, cognitive, or mechanical) [51]. The activation of the prefrontal cortex (PFC), auxiliary motor area (SMA), and premotor cortex (PMC) often occur in response to static balance challenges [53].

Using the star excursion balance test, previous research investigating the level of development of children’s dynamic balance discovered a difference between the test’s anterior direction and other directions. This difference was for both excursions of the contralateral leg in both genders. This finding might be explained by the location of the center of mass, which is altered by being pushed backward in the case of an anterior excursion, limiting the expression of balance, particularly in untrained children. [54].

Some comments on changes in functional performance indicate the impact of balance training on various sports and static postural swing and dynamic balance changes in nonathletes [55,56,57]. Compared with untrained control participants, nonathletes also improved their lower limb muscle strength after balance training. However, balance training is not as practical as strength training [11].

Other findings regarding assessing balance and proprioceptive control highlighted the idea that the RM and VM values of balance tests performed with eyes open are very similar across three groups: basketball players, swimming athletes, and the control group. This indicates that the postural control with eyes open does not appear to be influenced by the practice of sports under consideration in tests performed with eyes closed; however, there was a different response concerning the difficulties of the proposed test [58]. In our study, the statistically significant data identified in the two variants of the stork test (eyes closed/open) in the nondominant male foot (*p* = 0.0179) can be explained in terms of the much richer motor baggage of athletes and their increased ability to integrate sensory information. No statistically significant differences were obtained in the dominant segment (eyes closed/open) because the balance exercises are underrepresented in the training programs of both basketball players, where the emphasis is on team play, and swimmers, where the activity takes place in an aquatic environment.

While a common aim for athletes is to improve proprioception and stability management throughout the exercise, and there is growing testimony indicating that proactive exercises such as wobble panel training help to achieve this, there might also appear an essential hereditary mechanism proprioceptive capacity and control of balance [26]. This is probable to appear more noticeable in brilliant sportspeople aspiring to appear the topnotch leading, wherever there are immediately exhaustive preparation levels. According to Han et al. [26], it appears that ankle proprioception marks were found to not substantially correspond with a long time of preparation, indicating that biologically defined factors can restrict the amount of ankle proprioception enhancement associated with sports training [59].

The results of another study showed that children who had always exercised in a club environment during the three-year test showed a better level of coordination than children who participated only partially or did not participate in the club environment. In addition, stability further shows that consistent sports participation over time, changes, or lack of motor coordination have no substantial impact on the development of motor coordination [60].

Consistent research of 43 studies revealed better balance performances in adolescents compared to children irrespective of the variable considered and partially proved that girls display superior balance behaviors compared to same-aged boys [61]

The results we obtained highlight a statistically significant difference between students who practice sports and those who do not practice sports in the ruler drop test, nondominant hand, in favor of athletes, which confirms the hypothesis proposed (sports performance improves hand–eye control and proprioceptive control).

## 5. Conclusions

The results of the study highlight a significant difference in reaction speed and hand–eye coordination in the nondominant segment (ruler drop test, *p* = 0.0212) of children who performed performance sports compared to those who did not practice sports.

Regarding proprioceptive balance and control, the only statistically significant difference between the results of the two sexes was the stork test with closed eyes and the nondominant leg (*p* = 0.0057).

## 6. Study Limitations

There are three significant limitations of this study that could be addressed in future research. First, the study focused only on a school population aged 14–15 out of 5 school units in the city of Târgu Mures. The second limitation is the choice of exploratory research, which is why we are considering conducting a longitudinal study of the cross-sectional detriment. This would add value to the research, with the added ability to observe changes in psychomotor skills over time, as well as the factors that influence them. The third limitation, sample size, is the distribution by gender, only 33 females vs. 74 males.

## Figures and Tables

**Table 1:** Statistical comparison between the two genders.

	Female Gender (33) Mean ± SD (Median)	Male Gender (74) Mean ± SD (Median)	*p*-Value
Ruler Drop Test			
Dominant hand	18.7 ± 8.292 (18.00)	16.24 ± 7.346 (14.82)	0.0957
Nondominant hand	18.86 ± 6.909 (17.00)	16.89 ± 7.384 (15.89)	0.3297
Standing Stork (Eyes Closed)			
Dominant leg	3.642 ± 2.309 (3.25)	4.130 ± 3.469 (3.420)	0.5848
Nondominant leg	2.916 ± 1.385 (2.66)	4.926 ± 3.666 (4.015)	* 0.0057
Standing Stork (Opened Eyes)			
Dominant leg	3.691 ± 2.111 (3.19)	3.870 ± 3.060 (3.095)	0.8820
Nondominant leg	3.280 ± 1.425 (2.84)	3.816 ± 2.620 (3.030)	0.2715

Legend: * Mann-Whitney test.

**Table 2:** Statistical comparison between children who practice sports and those who do not practice.

	Practice Sport (47) Mean ± SD (Median)	Do Not Practice Sports (60) Mean ± SD (Median)	*p*-Value
Ruler Drop test			
Dominant hand	15.76 ± 6.822 (14.30)	17.95 ± 8.2436 (16.81)	0.1459
Nondominant hand	15.63 ± 7.682 (14.71)	18.41 ± 6.652 (17.56)	* 0.0212
Standing Stork (Eyes Closed)			
Dominant leg	4.065 ± 3.012 (3.46)	3.913 ± 3.284 (3.325)	0.8359
Nondominant leg	4.764 ± 3.997 (3.33)	3.947 ± 2.545 (3.115)	0.5937
Standing Stork (Opened Eyes)			
Dominant leg	3.957 ± 3.482 (3.15)	3.704 ± 2.129 (3.215)	0.4899
Nondominant leg	4.022 ± 2.779 (3.30)	3.360 ± 1.867 (2.865)	0.1368

Legend: * Mann-Whitney test.

**Table 3:** Analysis of the medians of the two tests, standing stork (eyes closed/opened) related to gender.

	Standing Stork (Eyes Closed) Mean ± SD (Median)	Standing Stork (Eyes Opened) Mean ± SD (Median)	*p*-Value
Dominant Leg			
Female gender (33)	3.642 ± 2.309 (3.25)	3.691 ± 2.111 (3.19)	0.7613
Male gender (74)	4.130 ± 3.469 (3.420)	3.870 ± 3.060 (3.095)	0.4588
Nondominant Leg			
Female gender (33)	2.916 ± 1.385 (2.66)	3.280 ± 1.425 (2.84)	0.2278
Male gender (74)	4.926 ± 3.666 (4.015)	3.816 ± 2.620 (3.030)	* 0.0179

Legend: * Wilcoxon test.

**Table 4:** Statistical correlation between the variation of the standing stork test.

Variables (*n* = 107)	Ruler Drop Test		
	r Coefficient	95% Confidence Interval	*p*-Value
Dominant Hand			
Standing stork (eyes closed)—dominant leg	−0.07377	−0.2600 to 0.1178	0.4502
Standing stork (eyes opened)—dominant leg	−0.03854	−0.2268 to 0.1525	0.6935
Nondominant Hand			
Standing stork (eyes closed)—nondominant leg	0.2294	0.04128 to 0.4018	* 0.0175
Standing stork (eyes opened)—nondominant leg	0.3256	0.1447 to 0.4855	* 0.0006

Legend: * Pearson correlation.

## Data Availability

Not applicable.
